# Case report: Chorea and cognitive decline in a young woman: instrumental and genetic assessment of a case originally diagnosed as multiple sclerosis

**DOI:** 10.3389/fgene.2023.1129289

**Published:** 2023-06-26

**Authors:** Clemente Dato, Emanuele Micaglio, Giada Moresco, Ornella Rondinone, Paolo Vitali, Carlo Pappone, Laura Fontana, Monica Miozzo, Luciano Bet

**Affiliations:** ^1^ Unit of Neurology and Stroke Unit, IRCCS Policlinico San Donato, Milan, Italy; ^2^Department of Neurology, Azienda Ospedaliera di Melegnano e Della Martesana, Melegnano, Italy; ^3^Department of Arrhythmology, IRCCS Policlinico San Donato, Milan, Italy; ^4^ Medical Genetics, Department of Health Sciences, Università Degli Studi di Milano, Milan, Italy; ^5^ Department of Biomedical Sciences for Health, University of Milan, Milan, Italy; ^6^ Unit of Radiology, IRCCS Policlinico, San Donato, Milan, Italy; ^7^ Medical Genetics Unit, ASST Santi Paolo e Carlo, Milan, Italy

**Keywords:** neuroinflammation, cognitive decline, chorea, multiple sclerosis, chorea (non-Huntington’s)

## Abstract

We describe the case of a young woman affected by debilitating chorea and rapidly progressive cognitive decline. While her original diagnosis was multiple sclerosis, we performed a full instrumental and genetic assessement, though which we identified multiple genetic variants, including a novel variant of the APP gene. We propose some possible mechanisms by which such variants may contribute to neuroinflammation and ultimately lead to this devastating clinical course.

## 1 Background

Multiple sclerosis (MS) is one of the most common diseases of the central nervous system (CNS). It affects more than 2.3 millions of people worldwide, mostly young adults and females, and is the most prominent cause of non-traumatic disability in this population (Filippi et al., 2018)⁠⁠. Its pathological hallmark is the demyelinating plaque, a focal area of inflammation localized in the white or grey matter.

We describe the case of a young woman affected by debilitating chorea and rapidly progressive cognitive decline. While her original diagnosis was multiple sclerosis, we performed a full instrumental and genetic assessment, though which we identified multiple genetic variants, including a novel variant of the APP gene. We propose some possible mechanisms by which such variants may contribute to neuroinflammation and lead to this devastating clinical course.

## 2 Case history and presentation

The patient was a 23-year-old young woman who used to work as an English teacher. Her family history was unremarkable. Her parents were not related; her mother was healthy, with no signs of neurological dysfunction; her father, though unavailable for examination and testing, was described as healthy. She was the younger of two siblings; her 33-year-old brother was healthy.

Her initial clinical history is fragmentary. At 21 years old, the patient presented with diplopia. She was diagnosed with MS in another center and treated with immunomodulating drugs, according to standard protocols. Her initial response to therapy was satisfactory. Over the following 2 years, however, her motor and cognitive condition progressively worsened. She became unable to continue her teaching activity and became bedridden.

At the age of 23, she came to our attention at the Emergency Department of San Donato Hospital (Milan), for the sudden onset of involuntary movements of the neck and limbs.

At examination, consciousness was preserved. Visual acuity and field of vision were normal; her gaze showed saccadic intrusions even in primary position. She was dysarthric and hypophonic. Her upper limbs were weak (4/5 on the Medical Research Council scale) and hypotonic. Her lower limbs were paretic (2/5 on the MRC scale) and spastic (3/4 on the modified Ashworth scale), with enhanced tendon reflexes; ankle clonus and Babinski sign were present bilaterally; she was bedridden and unable to adjust herself on the bed. Sensation was normal. Clinical picture was dominated by diffuse, twisting choreoathetosic movements of the neck and limbs, without significant lateralization.

She was able to say her name and speak with a basic vocabulary; she was able to follow simple directions, but comprehension was clearly limited. Cognitive examination was only possible through the use of the Severe Mini-Mental State Examination scale (Harrell et al., 2000 final score was 8/30, indicative of a state of dementia.

She was admitted to the Neurology unit. A full diagnostic evaluation with blood test, cerebrospinal fluid (CSF) analysis, instrumental imaging, and genetic analysis were performed.

Given the earlier diagnosis of MS, pulse therapy with methylprednisolone (1 gr per day for 5 days) was administered, without any effect. After that, the introduction of increasing dosages of tetrabenazine and baclofen led to satisfactory control of the involuntary movements. Tetrabenazine and baclofen are, respectively, a VMAT2 inhibitor and a GABA agonist that are a commonly employed in the treatment of involuntary movements, regardless of the causing mechanism.

No other changes to her neurological state were recorded during her hospital stay, which lasted approximately 1 month. She was later transferred to an assisted living facility.

## 3 Investigations and results

### 3.1 Serological tests and imaging

Extensive immunological analysis was performed, including tests for anti-streptolysin O, anti-endomysium IgA and IgG, anti-neutrophil cytoplastic (ANCA), anti-DNA, anti-smooth muscle, anti-nuclear antigens (ANA and ENA), anti-glutamate decarboxylase (GAD), anti-gliadin, anti-cardiolipin, anti-phospholipid, anti-thyroglobulin, anti-thyroperoxidase, anti-aquaporin-4, anti-myelin oligodendrocyte glycoprotein, all of which turned out negative. A panel for anti-NMDA receptor encephalitis and other forms of autoimmune encephalitis was negative. Anti-HIV 1, 2 antibodies were negative.

**FIGURE 1:**
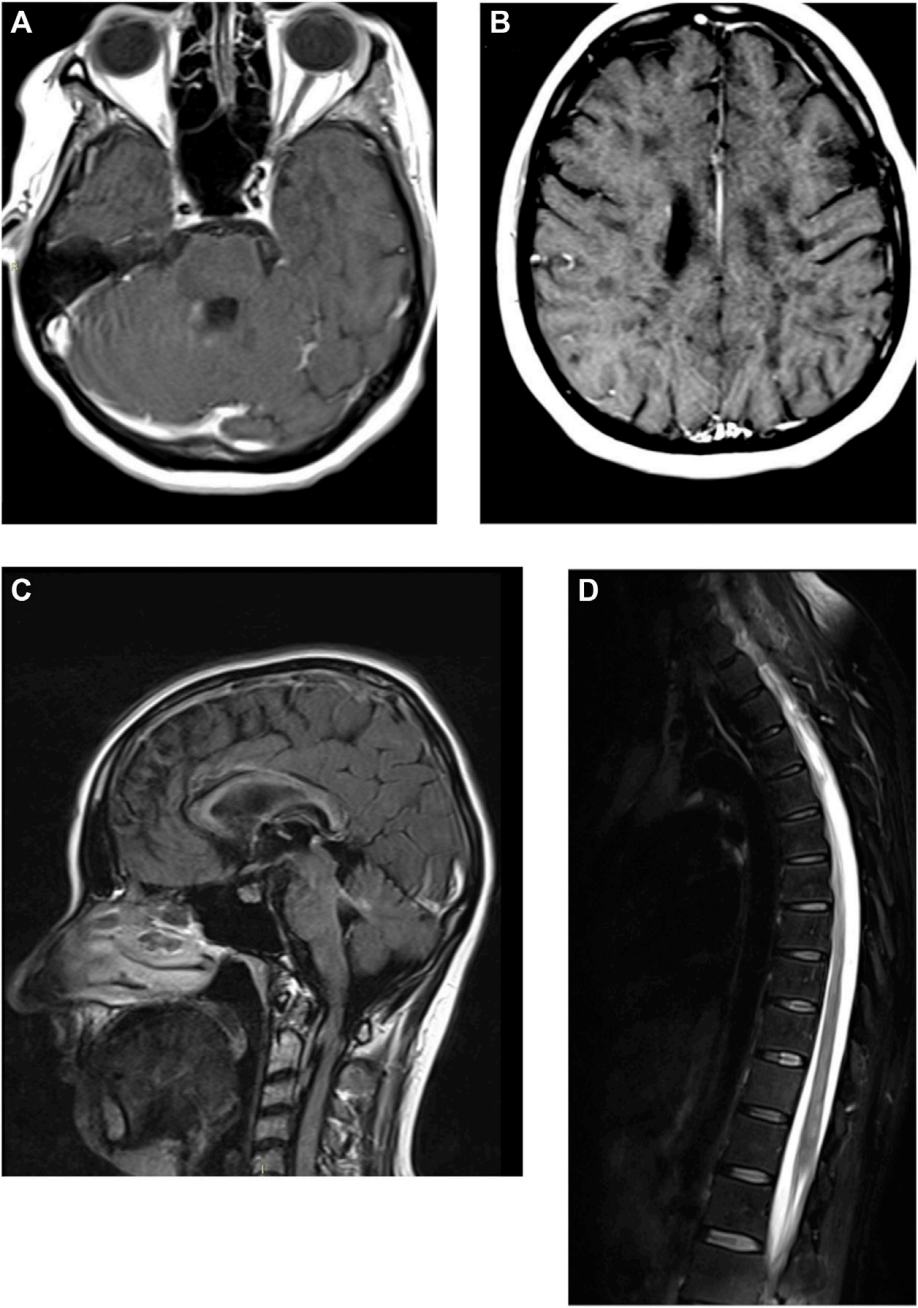
Brain and spine magnetic resonance imaging of the patient, with gadolinium enhancement. **(A, B)** T1-weighted axial images of the brain. **(C)** FLAIR sagittal image of the brain. **(D)** T2-weighted image of the dorsal spinal chord.

Brain MRI images were acquired on a Siemens 1,5T scanner with DWI, FLAIR, T1, T2* on axial planes, FLAIR on sagittal planes, T1 axial after gadolinium injection. Acquisition was partially hindered by the involuntary movements; due to blood pressure instability proper sedation was impossible. Brain MRI showed two focal lesions into the right cerebellar hemisphere, periventricular to the fourth ventricle, which showed contrast enhancement compatible with acute demyelinating lesions (Figure 1A). Fourth ventricle was slight dilated exvacuo. The supratentorial white matter was diffusely abnormal, with more focal periventricular lesions hypointense in T1 (back holes), mainly in the peritrigonal white matter (Figure 1B). Periventricular confluent lesions were also seen in sagittal FLAIR along the inner margin of the whole corpus callosum, diffusely thinned (Figure 1C). No supratentorial lesions showed contrast enhancement. Lateral ventricles were also significantly enlarged, including other temporal horns due temporomesial atrophy. Diffuse sulcal enlargement was most likely due to cortico-subcortical atrophy. Spine MRI was acquired by a second scan with stronger sedation, with T1, T2, T2 TIRM sagittal planes, T1 after gadolinium injection (Figure 1D). Spine MRI showed patchy confluent lesion within the spinal cord, without any evidence of contrast enhancement. The spinal cord showed also slight diffuse atrophy.

### 3.2 CSF analysis

Spinal fluid showed 30 mononucleated cells/mmc; total protein was 63,32 mg/dL, Link’s index was 2,54; multiple oligo clonal bands not present in serum were detected. Testing for antibodies associated with common causes of encephalitis was negative. CSF ELISA assay also revealed total tau (T-tau) 251 ng/L, phosphorylated tau (P-tau) 15.4 ng/L, Aβ42 212 ng/L, Aβ40 2,807 ng/L; Aβ42/tau ratio was 0.845, while Aβ42/P-Tau ratio was 12.766. Table 1 compares our results with the reference values in literature (Sjögren et al., 2001)⁠. Reference values for normal T-tau are age-dependent (Hampel and Blennow, 2004)⁠; in particular, between 21 and 50 years of age T-tau should be < 300 pg/mL. In summary, CSF assay revealed strikingly low values of Aβ42 and Aβ40, compared to healthy controls, with normal T-tau and P-tau values.

**TABLE 1:** CSF values detected in the patient compared to reference values.

	Reference values	Patient
CSF T-tau	<404 (<300) ng/L	251 ng/L
CSF P-tau	<56.5 ng/L	15.4 ng/L
Aβ42	>599 ng/L	212 ng/L
Aβ40	7,755–16,715 ng/L	2,807 ng/L
Aβ42/tau	>1.275	0.845
Aβ42/phospho tau	>8.100	12.766

### 3.3 IT15 gene testing

Testing for Huntingon’s disease turned out negative.

### 3.4 Whole exome sequencing

DNAs were extracted from peripheral blood sampled from the patient, her mother and her brother. WES was carried as previously described using the SureSelectQXT Clinical Research Exome V2 kit (Agilent, Santa Clara, CA) and sequenced using NextSeq 550 (Illumina, CA, United States). eVai software (enGenome, Pavia, Italy) and several *in silico* prediction tools (including SIFT, PolyPhen2, MutationTaster) were employed for variant classification and prioritization. Putative pathogenic variants identified by WES were subsequently confirmed by Sanger sequencing.

A specific informed consent for WES was obtained for all analyzed family members. The DNA sample from the father was not available.

WES identified 71,087 variants, with a Q30 sequencing percentage of 88,9% and uniform coverage above 100X. After the filtering steps, variant interpretation according to the clinical data allowed the identification of three candidate variants (Table 2).

**TABLE 2:** Details of identified candidate variants and classification according to the American College of Medical Genetics (ACMG) (Richards et al., 2015)⁠ All variants were detected in a heterozygous state.

Gene (RefSeq)	Variant (exon)	SNP ID (dbSNP)	Protein change	ACMG classification	Identified in
*APP* (NM_000484)	c.1726G>A (exon 14)	rs200769792	p.V576I	VUS: PM2, PM6, PP2, PP3, BS2	Patient only
*MPO* (NM_000250)	c.1555_1568del14 (exon 9)	rs536522394	p.M519Pfs*21	Pathogenic: PVS1, PM2, PP3	Patient and brother
*GAB2* (NM_080491)	c.1325G>A (exon 6)	rs202165383	p.R442Q	Likely benign: PM2, PM6, BS1, BP1	Patient only

VUS, variant of uncertain significance; PVS1, pathogenic, very strong (null variant); PM2, pathogenic moderate (absent from controls); PM6, pathogenic moderate (assumed *de novo*); PP2, pathogenic supporting (missense variant in a gene that has a low rate of benign missense variation); PP3, pathogenic supporting (multiple lines of computational evidence support a deleterious effect on the gene or gene product); BS1, benign strong (allele frequency is greater than expected for disorder); BS2, benign strong (observed in a healthy adult individual); BP1, benign supporting (missense variant in a gene for which primarily truncating variants are known to cause disease).

The novel missense variant c.1726G>A (p.V576I) in exon 14 of the APP gene (21q21.3) was identified in the patient only. This mutation is predicted to be damaging by nine out of thirteen *in silico* prediction tools, and this score is consistent with the amino acid substitution in a highly conserved residue.

The 14-bp deletion c.1555_1568del14 (p.M519Pfs*21) in exon 9 of the MPO gene (17q22) was also present in the healthy brother. This variant was classified as pathogenic; it causes a shift in the reading frame with the generation of a premature stop codon, leading to a truncated MPO-precursor lacking 228 amino acids. The ACMG classification of the MPO variant is referred to a homozygous state.

The novel missense variant c.1325G>A (p. R442Q) in exon 6 of the GAB2 gene (11q14.1), identified in the patient only and was classified as likely tolerated by most *in silico* pathogenicity predictors. Because of the lack of her father DNA, we were not able to classify it as a *de novo* variant. All candidate variants were confirmed by Sanger sequencing.

## 4 Discussion and conclusion

When the patient first came to our attention, a diagnosis of multiple sclerosis had already been made. The clinical presentation of MS is extremely heterogeneous, and it is closely dependant on the location of the demyelinating plaques; it may comprise sensory, motor, and cognitive symptoms. Onset of MS typically occurs in the third or fourth decade, and its course may be characterized by relapses and remissions or by a steady progression over the years (Filippi et al., 2018)⁠.

Regarding our case, radiological evidence of distinct lesions, and the presence of oligoclonal bands in the CSF could contribute to the diagnosis of MS, according to the 2017 revisions of the McDonald criteria for the diagnosis of MS (Thompson et al., 2018). However, several atypical features warranted further investigation.

Particularly striking was the rapid course of the disease, progressing from diplopia to a severe motor and cognitive impairment in only 3 years, without any clear evidence of distinct episodes. Cognitive impairment is a feature of late MS, occurring in 40%–70% of patients, and is related to damage to both white and grey matter (Rocca et al., 2015)⁠. MRI findings of this severity, with widespread white matter alterations and grey matter atrophy, are unusual in young patients with MS. Besides infective and inflammatory causes, other forms of rapidly progressive dementia, like Creutzfeldt-Jacob disease, were ruled out due to the initial response to immunomodulating drugs and the incompatible MRI findings.

Chorea and related movement disorders, such as hemiballismus and athetosis, are an unusual occurrence in MS as well; when they do occur, they are usually unilateral and associated with demyelinating lesions in the contralateral basal nuclei (Mehanna and Jankovic, 2013)⁠. In contrast, our patient had bilateral choreoathetosis without overt basal nuclear involvement. Other common causes of chorea, such as metabolic imbalances and deficiencies, polycythaemia, and connective tissue disorders, were excluded by laboratory testing.

Given these atypical symptoms, the chronic and progressive course and the absence of other elements that could explain neuroinflammation, we considered the possibility of an underlying genetic anomaly that could be responsible for the complex clinical picture seen in this patient.

Testing for Huntington’s disease, the most common cause of inherited chorea, turned out negative. We then performed WES to uncover the possible presence of genetic variants. Variant filtering and analysis according to the clinical data did not highlight a unique candidate variant with high confidence; we hypothesize that variants in several genes related to neurodegenerative conditions (APP, MPO, GAB2) could contribute to the severe phenotype of the patient.

Mutations in the APP gene are primarily associated with dominantly inherited forms of Alzheimer’s Disease (AD, OMIM #104300) and Cerebral Amyloid Angiopathy (CAA, OMIM #605714). Alzheimer’s disease (AD) is a neurodegenerative disease mainly characterized by progressive cognitive decline; while sporadic AD has its mean age of onset at 80 years, autosomal dominant inherited forms may present as early as in the fifth decade (Masters et al., 2015)⁠. Our patient does not satisfy the diagnostic criteria for Alzheimer’s disease (Cummings et al., 2013).⁠ Notably, AD has a characteristic CSF profile, with increased Aβ42 and high T-tau and P-tau levels. Our patient’s CSF profile, with decreased Aβ42 levels (and increased Aβ42/Aβ40 ratio), was consistent with those of pre-symptomatic AD patients (Toledo et al., 2013)⁠. Low levels of Aβ42 are also consistently found in the CSF of MS patients (especially in those with the relapsing-remitting and primary progressive forms) that respond to immunomodulating treatment (Mai et al., 2011; Augutis et al., 2013).⁠

The APP gene encodes a cell surface receptor involved in neurite growth, neuronal adhesion, axon genesis, synaptogenesis, cell mobility and transcription regulation. APP is cleaved by secretases to form several peptides; accumulation of these peptides, and particularly of Aβ42, is believed to be crucial in the pathogenesis of AD (Masters et al., 2015)⁠. Indeed, an increased ratio between Aβ42 peptides and the smaller Aβ40 (Aβ42/Aβ40) is a biomarker of early cognitive deterioration in patients with AD, and can be measured up to 15 years before the onset of dementia (Masters et al., 2015)⁠. The mutation type and associated Aβ42/Aβ40 ratio predict the mean age of onset of dementia, as confirmed in the Dominantly Inherited Alzheimer Network (DIAN) study: most pathogenic APP mutations alter APP processing, indirectly increasing the Aβ42/Aβ40 CSF ratio (Masters et al., 2015)⁠.

The 1726G>A variant detected in our patient is neither reported in ClinVar nor in HGMD databases and is predicted to be damaging by most *in silico* tools (Table 2). At the protein level, this variant (p.V576I) falls in the extracellular domain, in a position apparently not crucial for the proper protein function. However, the mutation may affect the function of an exonic splicing enhancer (ESE) mapping at the position c.1726, as predicted by the Human Splicing Finder *in silico* tool. The observed variant may thus alter APP mRNA splicing (Fig. X) leading to the skipping of exon 14 (amino acids 563–637) or the skipping of both exon 14 and 15 (amino acids 563–655). We were not able to verify this hypothesis, as the patient was unavailable for further testing.

An APP isoform lacking exon 15 (L-APP) is physiologically expressed in glial cells, but not in neurons. The deletion of exon 15 alters protein trafficking so that L-APP reaches both the basolateral and the apical surfaces, possibly leading to alternative APP cleavage events. Furthermore, the deletion of exon 15 results in the formation of a consensus sequence (Glu-Xaa-Ser-Gly) for the addition of chondroitin sulfate proteoglycan (CSPG), and also the skipping of both exons 14 and 15 (which could be the case of our patient) maintains a similar consensus sequence (Glu-Val-Val-Ser-Gly). Appicans are CSPG secreted and associated with cells that contain L-APP as their core protein, and they are physiologically produced by astrocytes. Since astrocytes are found associated with neuritic plaques and participate in the formation of brain scars following neuronal injury, astrocytic appicans may as well be involved in the development of pathological neuroinflammatory structures (Pangalos et al., 1995)⁠. Interestingly, CSPGs have been found in both senile plaques and neurofibrillary tangles (McGeer and McGeer, 1995)⁠. Thus, we hypothesize that the c.1726G>A variant might lead to skipping of the APP exons 14 and 15, thus increasing the secretion of appicans, from both astrocytes and neurons, resulting in an increased neuroinflammatory condition. This hypothesis is supported by the evidence of reduced CSF Aβ42 levels in the patient.

The heterozygous deletion c.1555_1568del14 identified in the MPO gene in the patient was probably inherited from the father, since the same variant is absent in the mother but present in the patient’s brother. The HGMD database reports this specific variant as a disease-causing mutation (DM) for Myeloperoxidase deficiency, which however follows an autosomal recessive inheritance. It is interesting to note that, in addition to myeloperoxidase deficiency, heterozygous mutations in the MPO gene are also associated with an increased susceptibility to juvenile-onset AD (OMIM #104300) and literature reports an association also with MS (Kantarci et al., 2000)⁠. However, the patient’s brother, who carries the same heterozygous mutation, is reported to be in good health. The MPO gene encodes for myeloperoxidase, which is part of the host defence system of polymorphonuclear leukocytes, and responsible for microbicidal activity against a wide range of organisms.

The same heterozygous deletion identified in the MPO gene was also described by Romano et al. (1997)⁠, who reported a case of a 5-year-old MPO-deficient subject whose father was also MPO-deficient (and carrier of two MPO mutations); no mutation was identified in the mother, even though she had 24% of normal MPO activity. Based on this report, we cannot exclude that the 14bp-deletion alone influences the activity of MPO. Although we unfortunately did not have the possibility to evaluate MPO activity in our patient, we cannot exclude that this variant might have contributed to the severe and rapidly progressive neuroinflammatory process described in our patient.

The missense variant c.1325G>A (p. R442Q) in the GAB2 gene was found in a heterozygous state only in the patient (absent in both her mother and brother). *In silico* prediction tools evaluated this variant as tolerated, but it has never been reported neither in Clinvar nor in HGMD databases. GAB2 is a member of the GRB2-associated binding protein (GAB) gene family, and it functions as adapter protein acting downstream of several membrane receptors including cytokine, antigen, hormone, cell matrix and growth factor receptors to regulate multiple signalling pathways.

Specific SNPs in the GAB2 gene have been associated with susceptibility to juvenile-onset AD (OMIM #104300). In particular, a recent study suggested that specific GAB2 variants were significantly associated with the level of the three CSF biomarkers of AD (Aβ, total tau and phospho-tau), further supporting a role of GAB2 in the modulation of AD risk (Chen et al., 2019).⁠ This evidence, coupled to the decreased Aβ levels found in the patient, sustain a possible additional effect of the GAB2 variant on the severe phenotype of the patient.

Taken together, these findings highlight that more than one variant could play a role in determining the severe and complex phenotype of the patient. Although a unique candidate variant with high confidence is not present, we cannot exclude that the contribution of variants observed in the APP, MPO and GAB2 genes related to neurodegenerative may account for the complex and severe phenotype of the patient. The frequent discovery of variants in several genes in the same patient, indeed, suggests reconsidering the genetic bases of complex phenotypes. We cannot exclude that a number of severe early-onset conditions may in fact be oligogenic, with satellite variations acting as modulators of the phenotype or resulting in new clinical entities.

## Data Availability

The datasets for this article are not publicly available due to concerns regarding participant/patient anonymity. Requests to access the datasets should be directed to the corresponding author.
